# Editorial: Opportunities and challenges of head and neck cancer treatment in the era of immune checkpoint inhibitors

**DOI:** 10.3389/fimmu.2025.1656915

**Published:** 2025-07-11

**Authors:** Zheran Liu, Lei Cai, Xingchen Peng

**Affiliations:** ^1^ Department of Biotherapy, Cancer Center, West China Hospital, Sichuan University, Chengdu, Sichuan, China; ^2^ Institute of Hepatopancreatobiliary Surgery, Chongqing General Hospital, Chongqing University, Chongqing, China

**Keywords:** head and neck cancer, immune checkpoint inhibitors, tumor micro environment, immunotherapy, cancer

Head and neck squamous cell carcinoma (HNSCC) remains one of the most challenging solid tumors to manage, with persistently suboptimal survival outcomes despite advancements in surgery, radiotherapy, and chemotherapy (1). Over the past decade, immune checkpoint inhibitors (ICIs)—particularly those targeting programmed cell death protein 1 (PD-1) and its ligand PD-L1—have revolutionized the treatment landscape of multiple malignancies, including HNSCC (2, 3). By reinvigorating cytotoxic T-cell responses, ICIs have demonstrated durable clinical benefits in subsets of patients with recurrent and metastatic disease, and in some contexts, have even redefined standards of care. Nevertheless, the response rates to ICIs in HNSCC remain limited to a fraction of patients, and the complexity of immune resistance, tumor heterogeneity, and adverse immune-related events pose significant barriers to universal efficacy (4, 5).

In this context, our Research Topic “*Opportunities and Challenges of Head and Neck Cancer Treatment in the Era of Immune Checkpoint Inhibitors*” was launched to provide a platform for cutting-edge research and clinical observations aimed at addressing these unresolved issues. The goal of this Research Topic is to deepen our understanding of immunotherapy mechanisms in HNSCC, highlight emerging biomarkers for patient stratification, explore innovative therapeutic combinations, and provide real-world insights into the optimization of treatment-related toxicity and efficacy. We believe this Topic provides a timely synthesis of current advancements and critical gaps in immuno-oncology as it relates to head and neck cancer.

A prominent theme in this Research Topic is the advancement of ICI-based neoadjuvant and multimodal therapies. Several studies examined the feasibility and efficacy of integrating ICIs with chemotherapy or radiotherapy in locally advanced HNSCC. Li et al. and Ding et al. demonstrated that neoadjuvant immunochemotherapy led to high rates of major or complete pathological responses in oral squamous cell carcinoma (OSCC), with improved locoregional control and survival. Yao et al. retrospectively compared PD-1 versus EGFR inhibitors in hypopharyngeal cancer, showing superior response and organ preservation in the PD-1 cohort. Sun et al. reported a case of paranasal sinus carcinoma achieving primary pathological response with neoadjuvant nivolumab and chemotherapy, providing clinical insight into rare subtypes. Yu et al. demonstrated that adding a PD-1 inhibitor to induction chemotherapy in nasopharyngeal carcinoma significantly improved complete response rates and survival outcomes. Collectively, these studies support the inclusion of ICIs in early-stage treatment, while highlighting the need for predictive biomarkers to guide patient selection.

Another key focus of this Research Topic lies in the discovery of predictive biomarkers and the application of computational approaches to inform immunotherapy strategies. He et al. applied multi-cohort transcriptomic analysis and machine learning to identify ten exosome-related genes relevant to immune evasion and prognosis, with ANGPTL1 showing promise as a novel biomarker. Tran et al. used electronic health record data and machine learning to detect ICI-induced inflammatory arthritis and associated immune-related adverse events, emphasizing the utility of data-driven approaches in toxicity prediction and management.

This Topic also features timely reviews addressing current challenges and therapeutic innovations. Aboaid et al. provided a comprehensive overview of immunotherapy trials in locally advanced and recurrent/metastatic HNSCC, including dual ICI combinations and novel agents such as virotherapy and CAR-T. Chen et al. synthesized meta-analysis data from randomized trials to compare ICI regimens in the first- and second-line settings for recurrent/metastatic HNSCC, with subgroup analyses based on PD-L1 expression. Their findings suggest that pembrolizumab combined with chemotherapy offers the most substantial PFS benefit among patients with high PD-L1 expression. Wu et al. reviewed small-molecule immunomodulators as potential adjuvants to overcome resistance, and Zhang et al. discussed the emerging application of NK cell therapies in OSCC, particularly CAR-NK technologies. Zheng et al. reviewed the immune microenvironment in papillary thyroid carcinoma, providing mechanistic insights relevant to immune modulation beyond classical HNSCC.

Real-world and rare case reports included in this Topic further illustrate the complexity of ICI-based treatment. Qing et al. described long-term immunotherapy with EBV-DNA monitoring in nasopharyngeal carcinoma, while Song et al. reported successful ICI rechallenge after anaphylaxis. Li et al. presented a case of sarcomatoid transformation following sequential ICI-targeted therapy, underscoring the importance of dynamic tumor profiling during treatment. Chen et al. analyzed thyroid dysfunction risks in patients receiving anti-PD-1 with or without radiotherapy, identifying key clinical factors associated with immune-related endocrinopathy.

Together, these contributions underscore both the promise and the complexity of immunotherapy in head and neck cancer. While ICIs offer durable responses and potential curative effects, their integration into multimodal regimens requires careful consideration of toxicity, resistance, and patient selection (6). Moving forward, the development of robust biomarkers, integration of multi-omics data, and application of AI-based tools will be essential to guide individualized therapy (7). This Research Topic highlights a dynamic and rapidly evolving field, and we hope it will inspire further translational efforts and collaborative innovation to optimize immunotherapy strategies for patients with HNSCC.
